# [*S,S*]-EDDS Ligand as a Soil Solubilizer of Fe, Mn, Zn, and Cu to Improve Plant Nutrition in Deficient Soils

**DOI:** 10.1021/acs.jafc.3c02057

**Published:** 2023-06-14

**Authors:** Sandra López-Rayo, Silvia Valverde, Juan José Lucena

**Affiliations:** Department of Agricultural Chemistry and Food Science, Universidad Autónoma de Madrid, 28049 Madrid, Spain

**Keywords:** ethylendiaminedisuccinic acid, biodegradable, fertilizer, calcareous soil, Phaseolus vulgaris

## Abstract

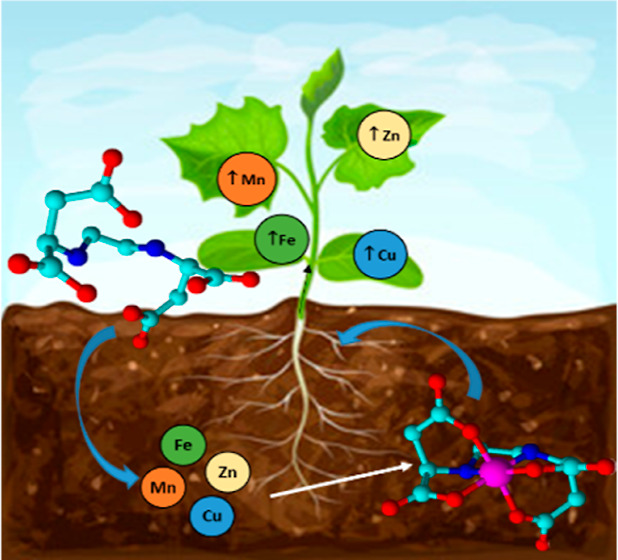

The deficiencies of iron, manganese, zinc, and copper in calcareous soils are a worldwide problem affecting plant growth and fruit quality, usually minimized by the application of recalcitrant synthetic metal chelates. Biodegradable ligand [*S,S*]-EDDS is an eco-friendly substitute. This study investigates the capacity of [*S,S*]-EDDS to mobilize micronutrients from agronomic soils and improve plant nutrition. A batch and a plant experiment (*Phaseolus vulgariscv*. Black Pole) with three agronomic soils was conducted to monitor the micronutrients solubilized by [*S,S*]-EDDS, the ligand degradation, and plant uptake. The results demonstrated the high capacity of [*S,S*]-EDDS to solubilize Fe and other micronutrients related to its chemical behavior and the enhancement of plant nutrition. The best results were shown in sandy-clay soil with low Fe, typically found in the Mediterranean areas. The results support the direct application of the ligand to soils and a possible biotechnological application of the ligand-producer bacteria.

## 1 Introduction

Most nutritional disorders in crops are associated specifically with deficiencies in micronutrients, such as iron (Fe), manganese (Mn), zinc (Zn) and copper (Cu).^1^ In general, Fe, Mn, and Cu deficiencies impair photosynthesis while Zn plays a role in meristematic growth. Several enzyme systems are regulated by micronutrients causing an important impact on flowering and fruit quality. Despite the fact that micronutrients are required in small quantities, their deficiencies are very frequent in calcareous soils and soil-less crops, usually in areas with alkaline pH, where these elements are not available for plants. Calcareous soils are common in the arid areas of the Earth^2^ occupying more than 30 percent of the Earth’s surface, including the Mediterranean basin, and comprising more than half of the agricultural soils in Spain. These soils are characterized by a high concentration of carbonates and bicarbonates that cause a buffering effect, setting the pH in a range of 7.5–8.5, inducing the deficiency of micronutrients in crops. This fact may be due to the lack of micronutrients or the formation of insoluble oxides being able to precipitate and be adsorbed^3^ causing a delay in plant growth, low production, and even death.

**Figure 1:**
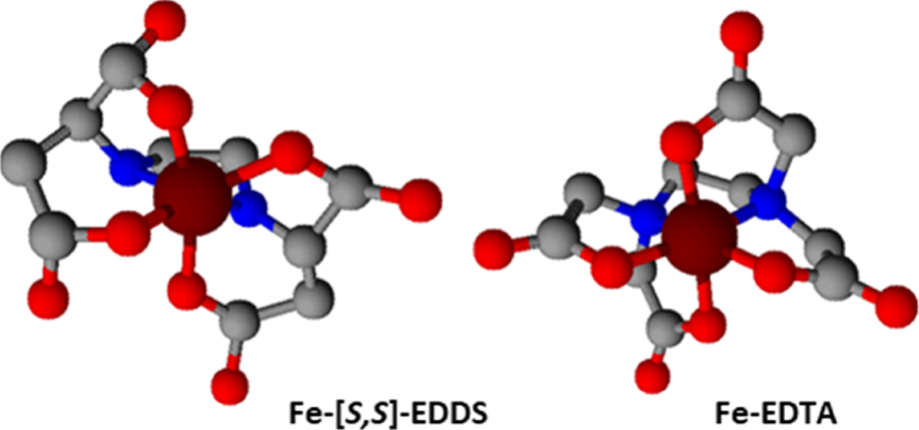
Chemical structure of the Fe chelates of [*S,S*]-EDDS and EDTA.

The most widespread agricultural practice to supply micronutrients is the application of fertilizers, which are conveniently supplied through fertigation, i.e., dissolved in the irrigation water.^4^ Micronutrient fertilization is traditionally done employing inorganic salts and synthetic chelates. The latter contains an organic molecule described as a chelating agent or ligand, which binds to the micronutrient metals, such as Fe, Zn, Mn, and Cu stabilizing them to a greater or lesser degree and, thus, making them available to the plant. While polyaminocarboxylic acids containing phenolate groups, such as ethylenediamine-*N*-*N*′bis(*o*-hydroxyphenylacetic) acid (*o*,*o*-EDDHA), are the most efficient chelating agents that provide Fe directly for soil application; the most commonly used chelating agents to apply various metal micronutrient solutions in low–medium reactive soils are ethylenediaminetetraacetic acid (EDTA), hydroxyethylenediaminetriacetic acid (HEEDTA), and diethylenetriaminepentaacetic acid (DTPA). They are amino polycarboxylic acids that form hexadentate complexes with bivalent and trivalent metal ions.^5^ The synthetic nature and the high durability of these chelating agents are responsible for their highly recalcitrant effect on the soil.^6,7^ The use of these synthetic chelates may represent an environmental risk due to their persistence and may lead to the solubilization of heavy metals naturally present in soils.^8^ In the last decade, efforts have been done by the scientific and agronomical communities to the development of a new generation of ligands with less impact on the environment to minimize their potential risks in soils.^9^ Here, naturally produced and biodegradable ligands represent the best alternative to traditional chelates, such as EDTA. The *S,S*′ isomer of the ethylenediaminedisuccinate (EDDS) is a chelating agent naturally produced by the actinomycete *Amycolatopsis japonicum* and a safe environmentally friendly substitute for EDTA (see Figure 1). The bacteria produce exclusively the [*S,S*] isomer under Zn-deficient growing media as a biological mechanism to improve the Zn solubility by its complexation.^10^ Studies showed that [*S,S*]-EDDS can be easily degraded to *N*-(2-aminoethyl) aspartic acid and, then, to ethylenediamine.^5,11^ In the presence of microorganisms, the final degradation products of [*S,S*]-EDDS are carbon dioxide and ammonium, the latter being useful to contribute to greater plant biomass.^12^ In addition, it should take into consideration that [*S,S*]-EDDS is less toxic to the microorganisms present in the soil, causing less stress.^13^

The advantages of [*S,S*]-EDDS in agriculture have been proven in several applications; the most widespread is metal phytoextraction from contaminated soils.^13−19^ Another potential application and less studied is as a fertilizer with a similar understanding, the [*S,S*]-EDDS could also mobilize metal micronutrients in soils (Fe, Mn, and Zn), improving crop nutrition in soils.

Previous studies have demonstrated that the application of [*S,S*]-EDDS complexed with Fe (as a Fe-[*S,S*]-EDDS chelate) improves the Fe nutrition in deficient plants growing under hydroponics and low-reactive growing media^20−24^ and also when applied as a foliar spray.^25^ More recently, the efficacy of the Fe-[*S,S*]-EDDS to cure Fe chlorosis has also been demonstrated in calcareous soils, presenting a higher efficacy than Fe-EDTA.^9^

Other studies have been oriented on the Zn and Cu complexation capacity of [*S,S*]-EDDS in agronomic conditions. For instance, some studies have shown the ability of [*S,S*]-EDDS mobilizing Zn in weakly acidic soil,^26−29^ and the efficiency of this chelate to improve the Zn nutrition in soybean plants applied as a micronutrient mixture (Zn, Mn, and Cu) chelated in basic pH-hydroponics and calcareous soil.^4,9,30−32^

Despite the fact that biodegradation of the [*S,S*]-EDDS limits its permanence in the soil, in contrast to the EDTA, its low affinity for calcium represents the main advantage of its effectiveness in calcareous soils. The speciation studies have already described that despite the lower values of the Fe, Mn, Zn, and, Cu stability constants of [*S,S*]-EDDS, in comparison to those of EDTA, the low interaction existing with Ca is responsible for the stability of Fe and other micronutrient metal chelates in calcareous soils.^9,31−34^

The current trends in fertilization supports biotechnological development, such as the use of beneficial microorganisms and their secretions. Under this context, the application of ligands naturally produced by bacteria or directly the application of the bacteria, such as the [*S,S*]-EDDS and the *A. japonicum*, can be an environmentally friendly approach for the nutritional improvement of crops grown in soils with immobilized micronutrients. The application of the ligand (without the micronutrients in the formulation) could also be a promising strategy in calcareous soils to mobilize micronutrients and, thus, improve plant nutrition and development in calcareous soils characterized by low-available micronutrient concentrations. Besides, this study is also applicable to the understanding of a future direct application of the [*S,S*]-EDDS bacteria producer to soils, which would work as a biofertilizer, naturally, and continuously producing this ligand.

Considering the proposed strategies, this study is aimed to investigate the capacity of the [*S,S*]-EDDS ligand to mobilize Fe, Mn, Zn, and Cu from agronomic soils to improve the nutrition of plants. With this objective, the solubilization of these micronutrients by [*S,S*]-EDDS and the ligand degradation were evaluated in three agronomic soils in batch experiments by a kinetic study and compared to EDTA. Then, the nutrient uptake by bean plants (*Phaseolus vulgariscv*. Black Pole) growing in a mixture of the aforementioned soils with calcareous sand was evaluated after application of [*S,S*]-EDDS.

## 2 Materials and Methods

### 2.1 Soil Properties

Three different agronomic Spanish soils were used for this study: S_1_, a sandy clay soil from Picassent (Valencia); S_2_, a clay soil from Alicante; and S_3_, a sandy loam soil from Burgos. The main characteristics of the soils are given in Table 1.

**Table 1:** Chemical and Physical Characteristics of the Soils (S_1_, Picassent Soil; S_2_, Alicante Soil; S_3_, and Burgos Soil)

	S_1_	S_2_	S_3_
sand (g Kg^–1^)	450	196	620
silt (g Kg^–1^)	50	396	220
clay (g Kg^–1^)	500	407	160
texture	sandy clay	clay	sandy loam
pH (H_2_O)	7.70	8.50	8.02
O.M. oxidizable (g·Kg^–1^)	9.2	19.4	7.6
CaCO_3_ total (g·Kg^–1^)	380	509	8.7
active lime (g·Kg^–1^)	89	226	1.1
micronutrient (Lindsay and Norvel (1978), mg·Kg^–1^)			
Fe	1.21	4.54	3.21
Mn	0.19	3.09	2.81
Zn	0.56	0.46	<0.2
Cu	0.87	2.76	0.92

### 2.2 Soil Batch Experiment: Interaction of Ligand Solutions with Soil Suspensions

A batch experiment was conducted with soil suspensions (in 1:5 soil: solution ratio) with three soils, as described in Section 2.1 to evaluate the metal solubilization and the [*S,S*]-EDDS degradation. A comparison with EDTA was done. For that, a modified methodology as that described by López-Rayo et al.^35^ was applied.

Briefly, 12.5 milliliters of ligand solution containing approximately 1 mmol L^–1^ [*S,S*]-EDDS (Na_3_[*S,S*]-EDDS solution 35%, w/w, Sigma-Aldrich, Darmstadt, Germany) or EDTA (Na_2_-EDTA, Titriplex III, 99.9%, w/w, Merck, Barcelona, Spain), and 12.5 milliliters of a mixture of 0.02 mol L^–1^ of 4-(2-hydroxyethyl)-1-piperazineethanesulfonic acid (HEPES) buffer (pH 7.5) and 0.020 mol L^–1^ of CaCl_2_ were added to 5.0 g of soil, previously sieved with a 2 mm mesh screen, in 60 mL closed plastic containers. The mixture was shaken in an orbital incubator (Boxcult J.P. Selecta, Barcelona, Spain) to allow the interaction for different times (3, 8, 24, 72, 168, 336, and 480 h) at 25 °C and 56 oscillations per minute protected from light exposure to avoid photodegradation. Soil solution samples were collected after the mentioned periods and filtered through syringe filters (nylon 0.45 μm, Labbox Labware S.L., Barcelona, Spain). Then, the pH was measured using an Orion Research Ion Analyzer EA920 (Orion Research, Franklin MA, USA). The concentration of Fe, Mn, Zn, and Cu in filtered solutions was determined by flame atomic absorption spectrophotometry (AAS) (Perkin-Elmer AAnalyst 800 spectrophotometer, Waltham, MA, USA) after an acidification of 5.0 mL of the filtrate with 0.5 mL of HCl 6.0 M (30% Suprapur, Merck, Germany). The ligand concentration in filtered solutions was monitored by high-performance liquid chromatography coupled to a photodiode array (HPLC-PDA) on a 2695 Waters HPLC system (Milford, MA, USA). Empower software (v 2.04) was used for system control and data acquisition. A symmetry C_18_, column 150 × 3.9 mm, 5 μm (Waters, Milford MA, USA) was employed for the determination of both ligands EDTA^36^, and the [*S,S*]-EDDS.^37^ The eluent for the [*S,S*]-EDDS method was composed of 5 mM of copper acetate monohydrate, 0.75 mM tetrabutylammonium hydroxide solution (TBAOH), and 4% of methanol (96:4, v/v) at pH 2.8, applied at a flow rate of 9.5 mL/min in the isocratic elution mode. For the EDTA determination, the mobile phase was composed of a mixture of 15 mM TBAOH at pH 6.0 and 30% of acetonitrile in water (70:30, v/v) applied at a flow rate of 1.5 mL/min in the isocratic mode.

The concentration of the ligands was monitored for 480 h (20 days) under controlled conditions described above. Ligand degradation was calculated according to eq 1
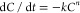
1where *n* is the order of the reaction. The goodness of fit for each kinetic mathematical model was evaluated by *R*^2^. Kinetic models were set up based on the analysis of experimental concentrations over time. The initial concentrations of ligands were considered as zero time (*C*_0_) to study the stability and degradation of the ligands. Kinetic parameters for the stability of the ligands, such as the rate constant (*k*, μmol·L^–1^·h^–1^ for order *n* = 0 kinetics and h^–1^ for order *n* = 1) and the half-life (*t*_1/2_, h) were determined. Kinetic rate constant (*k*) refers to degradation and the half-life (*t*_1/2_) is the time taken for a certain amount of ligand (*C*_0_) to be reduced by half.

### 2.3 Plant Experiment: Nutrient Uptake Improvement after Ligand Solubilization

A plant pot experiment was conducted to evaluate the nutritional status improvement (focused on the micronutrients Fe, Mn, Zn, and Cu) of bean plants (*P. vulgariscv*. Black Pole) growing in soil media containing a mixture of calcareous sand and individual agronomic soils described above, after the application of the free ligand [*S,S*]-EDDS. A comparison was done with plants growing on the same soil media but without any ligand application.

The experiment was carried out under controlled conditions of temperature and relative humidity (25 °C/40%/16 h day and 22 °C/60%/8 h night) in a Dycometal-type CCK model CCKF 0/16985 growth chamber provided with fluorescent and sodium vapor lamps.

Seeds were germinated using a standard seed-growing procedure. The seeds were washed with distilled water for 30 min and then placed in closed sterilized trays between cellulose paper sheets soaked with distilled water for 4 days at 30 °C in the darkness. Seedlings of similar development were placed on a holed plate floating over containers with a 5 L diluted nutrient solution buffered at pH 6.5 with the following nutritional composition: 0.04 mM Ca(NO_3_)_2_; 0.036 mM KNO_3_; 0.012 mM MgSO_4_·7H_2_O; 0.004 mM KH_2_PO_4_; 1.4 μM NaCl; 0.4 μM H_3_BO_3_; 0.002 μM Na_2_MoO_4_·2H_2_O; 4.6 μM Na_2_·EDTA; 0.1 μM MnSO_4_·H_2_O; 0.04 μM CuSO_4_·5H_2_O; 0.4 μM ZnSO_4_·7H_2_O; 0.04 μM NiCl_2_·6H_2_O; 0.04 μM CoSO_4_·H_2_O; and 0.2 μM Fe-HBED (*N*,*N*′-Bis(2-hydroxybenzyl)ethylenediamine-*N*,*N*′-diacetic acid). After 4 days, the nutrient solution was replaced to increase the nutrient concentration while removing Zn, Mn, and Fe to induce these micronutrient deficiencies with the following nutrient solution composition: 0.2 mM Ca(NO_3_)_2_; 0.18 mM KNO_3_; 0,18 mM MgSO_4_·7H_2_O; 0.02 mM KH_2_PO_4_; 7 μM NaCl; 2 μM H_3_BO_3_; 0.01 μM Na_2_MoO_4_·2H_2_O; and 23 μM Na_2_·EDTA. One gram of CaCO_3_ was added to each container buffering the pH to 8.2 to simulate calcareous conditions on the growing media. Four days later, the deficient plants were transferred to pots with the soil-growing media.

Previously, pots of 0.5 L volume (7 cm diameter and 16 cm high methacrylate cylinders) were filled with 0.42 kg of the corresponding soil (S_1_, S_2_, or S_3_, described in Table 1) sieved at 3 mm and 0.18 kg of sand (975 g Kg^–1^ CaCO_3_, 1–3 mm size) in a 70:30 soil: sand ratio. Two days before transplanting, the pots were irrigated with water until 100% of the soil–sand mixture water-holding capacity (136 mL for S_2_ and 96 mL for S_1_ and S_3_). For the rest of the experiment, the pots were kept at 80% of the maximum water-holding capacity. Daily irrigation was done by the alternative application of pure water or a macronutrient solution with a composition of 0.2 mM Ca(NO_3_)_2_; 0.18 mM KNO_3_; 0.18 mM MgSO_4_·7H_2_O; 0.02 mM KH_2_PO_4_ buffered at pH 8.2 with 0.1 g L^–1^ of CaCO_3_ and 0.1 g L^–1^ of NaHCO_3_ to simulate carbonated irrigation waters, similar to those typically found in Mediterranean agronomic areas. A Petri plate was placed under each pot to control possible leaching. Two seedlings were transferred to each pot.

The application of [*S,S*]-EDDS ligand was done through a solution previously prepared and applied to the top of the pot. The total dose was 16.8 μmol pot^–1^, split into six applications of 2.8 μmol pot^–1^ (three times the first week, twice the second week, and once the third week; designed as days 0, 3, 7, 11, 14, and 17).

#### 2.3.1 Plant Material Analysis

During the experiment, the leaf chlorophyll index was recorded by a Minolta SPAD-502 chlorophyll meter (Minolta, Osaka, Japan; three measurements per plant level). Twenty-one days after the first application of the ligand solution (day 0), the plants and the soil were harvested. For that, leaves, stems, and roots were separated and washed with 0.1% (w/v) HCl and 0.01% (w/v) non-ionic detergent solution (Tween 80, Probus, Barcelona, Spain) to remove any inorganic surface deposits and rinsed twice with ultrapure water. The samples were then dried in a forced air oven at 65 °C for 3 days, weighed, and milled in a porcelain mortar. Homogenized dried samples were digested first in a muffle furnace for dry digestion at 480 °C for 4 h, followed by acid digestion with 1:1 diluted HCl (Suprapur) for ash solubilization in a heating plate at 80 °C (Jones, 2001). Finally, the total Fe, Mn, Zn, and Cu concentration in the digested samples was determined by AAS after filtration through 20–25 μm pore paper filters (FilterLab 1238).

#### 2.3.2 Soil Analysis

The Fe, Mn, Zn, and Cu soluble and available fractions in the remaining soil were determined. For that, the soil content of each pot was collected and homogenized in a plastic bag. Two subsamples of 20 g were taken and extracted with 15 mL of ultrapure water, shaken for 60 min, centrifuged for 10 min at 6000 cycles min^–1^, and filtered through 20–25 μm pore paper filters to obtain the soluble fraction solution. The remaining soil was then shaken with 20 mL of a diethylenetriaminepentaacetic acid–triethanolamine solution (DTPA–TEA)^38^ for 60 min, centrifuged for 7 min at 9000 cycles min^–1^, and filtered through 20–25 μm pore paper filters to obtain the available fraction solution. The samples were finally analyzed for the Fe, Mn, Zn, and Cu concentrations by AAS after their acidification with HCl.

#### 2.3.3 Statistical Analysis

The general linear model procedure in Statgraphics Plus 5.1 (StatPoint, 2000) was used to compare the three factors (soil type, treatment, and day) for the SPAD values, and two factors (soil type and treatment) on the rest of the variables measured in the plant experiment.

## 3 Results and Discussion

### 3.1 Fe, Mn, Zn, and Cu Solubilization from the Soil by [*S,S*]-EDDS and EDTA Ligands

**Figure 2:**
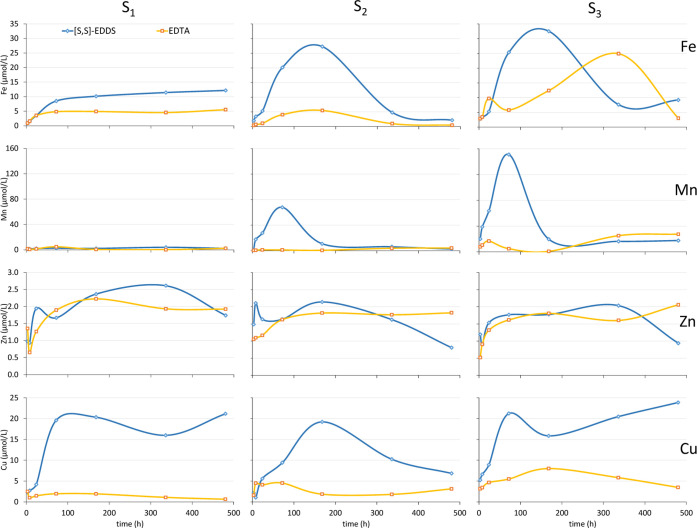
Fe, Mn, Zn, and Cu concentrations (μmol L^–1^) in the solutions at different interaction times with three calcareous soils from the soil incubation experiment. Values are means (*n* = 3).

The solutions obtained after the batch incubation of the three agricultural soils with ligand solutions at 0.500 mmol/L EDTA or 0.401 mmol/L [*S,S*]-EDDS were analyzed to determine the micronutrient solubilization through a kinetic study. The pH of the solutions was in the range of 6.9–7.4, thus, little differences can be expected due to pH variations according to the metal chelate speciation at this pH range.^9,30,39^ The Fe, Mn, Zn, and Cu concentrations in the solution obtained at different times are represented in Figure 2. In general, higher solubilization was achieved by the [*S,S*]-EDDS in the three soils investigated. Only in the Zn analysis, the results obtained for both ligands were similar, not only in the kinetic behavior but also comparing the three soils. Focusing on the differences observed for each metal, it must be noted that the micronutrient-available concentration (DTPA–TEA extraction), according to the soil characterization was different and thus, the maximum solubilization capacity of the ligands too. For a better understanding, a comparison of the naturally available micronutrient concentrations in soils and the maximum solubilization achieved by the ligand solutions (calculated at the same solution/soil ratio and expressed in μmol/L) is presented in Table 2.

**Table 2:** Maximum Soluble Micronutrient Concentrations in Soil Extracts Obtained in the Soil Batch Experiment with Both Ligands ([*S,S*]-EDDS and EDTA)^a^

	S_1_			S_2_				S_3_				
	Fe	Mn	Zn	Cu	Fe	Mn	Zn	Cu	Fe	Mn	Zn	Cu
maximum solubilized metal with [*S,S*]-EDDS (μmol/L)	12.1	3.94	2.61	20.3	27.3	68.0	2.04	19.2	32.6	151	2.03	21.2
maximum solubilized metal with EDTA (μmol/L)	5.52	4.88	2.23	2.36	5.45	4.08	1.82	4.53	24.9	27.4	2.06	8.00
available in soil (μmol/L)	4.34	0.69	1.70	2.73	16.3	11.2	1.40	8.68	11.5	10.2	0.61	2.90

a Values are means ± SD (*n* = 3). The [*S,S*]-EDDS and EDTA were added at measured concentrations of 401 and 500 μM. For comparison also the calculated concentration of the initially available metals in each soil extract is presented.

Soluble concentrations of Fe and Mn after solubilization by [*S,S*]-EDDS presented a similar trend with S_2_ and S_3_, reaching the maximum in the first period studied. For Mn, a quick decrease was observed immediately after the maximum while for Fe, the decrease was more gradual in time. In terms of the total metal solubilized from soils, the Fe concentration was increased by 1.7–2.8 fold, and Mn by 6–15 fold compared to the available metals in soils (Table 2). This means that the [*S,S*]-EDDS not only mobilizes the available fraction but also a more retained fraction of metals in the soil, especially in the case of Mn. However, this Mn in solution is transformed into insoluble forms in a short time. Redox conditions can also affect the dynamics of Mn because of the experimental design. The solutions were put in contact with dry soils; thus, a drop in the redox potential is expected during the first hours, due to increasing microorganism activity, improving the solubility of Mn and Fe.^40^ As mentioned, S_1_ presented a different profile, showing lower and slower solubilization. The naturally available Fe and Mn in this soil were remarkably lower than in the others. Here, a clear maximum solubilization value for Mn and Fe was not reached at the time assayed, but in any case, the concentrations found were significantly higher than those naturally available in the soil. It is notable, that in general EDTA solubilized significantly lower concentrations of Fe and Mn in the soils. Natural Mn available in S_1_ was remarkably low, so the Mn solubilization by both ligands, in this case, was scarce.

As mentioned above, the Zn solubilization by [*S,S*]-EDDS and EDTA was similar. The three soils presented very low Zn available concentrations (equivalent to 0.61–1.70 μmol/L, see Table 2). Interestingly, Zn solubilized was maintained for 360 h, suggesting that the Zn chelated by [*S,S*]-EDDS or EDTA was not sensitive to transformation into insoluble forms. This Zn-EDTA stability is already known in agriculture, being an efficient choice for Zn fertilization. The high stability of the Zn-[*S,S*]-EDDS chelate has been described in several soil conditions.^26−29^ The lesser biodegradability of [*S,S*]-EDDS/Zn than other metal [*S,S*]-EDDS chelates^41^ may also contribute to explaining these results.

The addition of EDTA increased slightly the concentrations of soluble metals in S_1_ and S_3_ (Table 2). However, in S_2_ there is almost no solubilization of micronutrients, which can be due to the physicochemical properties of this soil. It is characterized by a high pH value (8.5) and a high concentration of CaCO_3_. It should be noted that at this pH, the Fe and Mn would be replaced by calcium in the EDTA chelate.^42^ This competence is not expected for [*S,S*]-EDDS due to its lower affinity to Ca already mentioned.^9,30−33^

Concerning Cu, the [*S,S*]-EDDS solubilized higher amounts of Cu than EDTA in all soils, being 7–10 fold larger than the naturally available Cu in soil, and only 0.5 to 2.4 fold in the case of EDTA (Table 2). For S_1_ and S_3_, a similar trend with time was observed (Figure 2), the Cu concentrations were continuously increasing, indicating that the maximum solubilization value was not reached after 20 days, suggesting that even more Cu can be mobilized for a longer time. For S_2_, having a higher pH, this increase in solubilization was for a shorter period. The stability constants of Cu with EDTA and [*S,S*]-EDDS are similar, which is different with the other metals,^33,43^ and contributes highly to solubilizing Cu.

Considering the metal concentrations analyzed in the soil solution samples, the sum of the micronutrients solubilized is far from reaching the total [*S,S*]-EDDS added, which means that possible chemical competencies due to the differences in their stability constants were not affecting; however, and as mentioned before, the presence of Ca, as a major component in soil solution, can largely affect metal solubilization in the case of the EDTA samples, due to its high affinity, and with less magnitude in the case of the [*S,S*]-EDDS, with a low affinity for Ca which has already been discussed.

**Figure 3:**
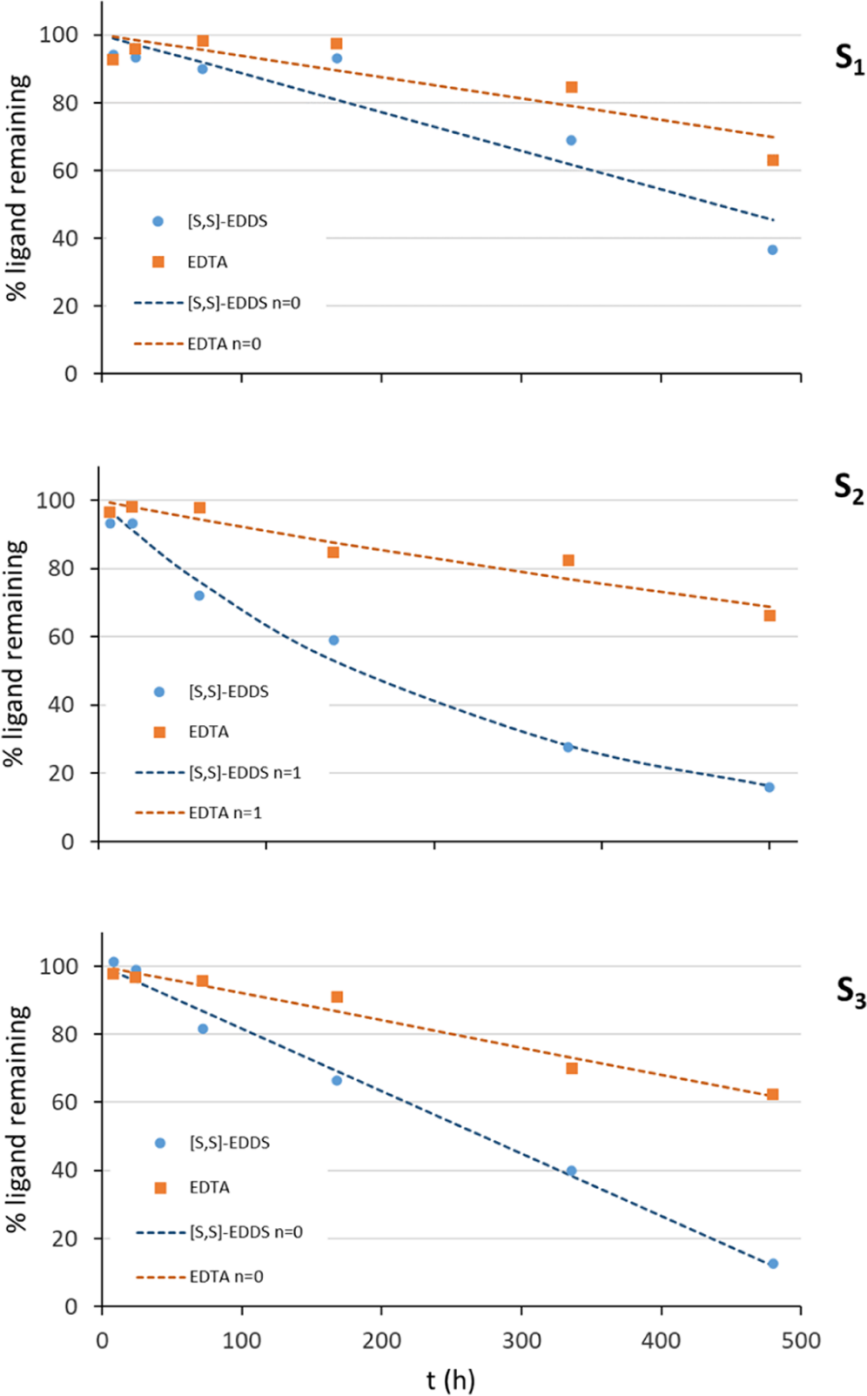
Percentage of ligand remaining in solution ([*S,S*]-EDDS and EDTA) at different interaction times with three calcareous soils from the soil incubation experiment. Dots represent mean values of experimental data (*n* = 3) and lines represent the kinetic linear fitting curves (kinetic parameters described in Table 3).

In addition to the metal concentration analysis, the concentrations of ligands were measured at different time intervals to study their stability. Figure 3 shows the percentage of chelating agents remaining in solution in the three soils during the time of the experiment, and the kinetic parameters obtained for n-order regression adjustment are described in Table 3. The ligand decrease in solution may be due mainly to both retention of their chemical species in the soil surfaces and biodegradation. Because EDTA slowly degraded, its results should show the retention of the ligand or chelation over time. For [*S,S*]-EDDS, the biodegradation is relevant, so its decrease in solution should be the sum of both processes. In fact, it was higher in the three soils than in EDTA. The mathematical model that best described both ligands’ stability in S_1_ and S_3_ was the zero-order, while a first-order fitted better to explain the stability in S_2_. It must be noticed that the regression coefficients for S_1_ obtained by the adjustment of the experimental data to zero-order and first-order were for both ligands similar to and lower than 0.9, but finally, the zero-order was chosen because of the higher R^2^ obtained. The first-order reaction is a reaction that proceeds at a rate that depends linearly on only one reactant concentration. Thus, ln(*C*_*i*_) is a linear function of time (h), *k* is obtained by data fitting, and *t*_1/2_ is determined as eq 2
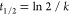
2

**Table 3:** Summary of the Kinetic Linear Fitting Parameters, *n*-Kinetic Order Reaction, *R*^2^-Coefficient of Determination, *k*-Rate Constant, and *t*_1/2_-Half-Life Corresponding to the Ligands Stability Obtained at Different Interaction Times with Three Calcareous Soils from the Soil Incubation Experiment

soil	ligand	*n*	*R*^2^	*k*	*t*_1/2_ (h)	*t*_1/2_ (days)
S_1_	[*S,S*]-EDDS	0	0.8790	0.00048	439	18
	EDTA	0	0.8755	0.00033	799	33
S2	[*S,S*]-EDDS	1	0.9933	0.00377	184	8
	EDTA	1	0.9226	0.00078	891	37
S3	[*S,S*]-EDDS	0	0.9905	0.00062	273	11
	EDTA	0	0.9687	0.00043	627	26

The *k* indicates a higher rate of degradation for [*S,S*]-EDDS and thus, *t*_1/2_ was significantly lower for EDTA. In this soil (S_2_), the differences between the two ligands were important. The zero-order reaction kinetics model fitted better to the stability data in S_1_ and S_3_, indicating that biodegradation and retention rates are independent of the ligand concentration. In this case, plotting *C*_*i*_ as a function of time, a straight line with a slope equal to *k* is obtained. For zero-order kinetics, *t*_1/2_ depends on *C*_0_ and *k*, as expressed by the equation
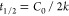


The kinetic constants were in this model similar for both ligands and soils but the durability of the ligand in solution was higher in S_1_, expressed by higher *t*_1/2_.

It is remarkable that less than 20% of the [*S,S*]-EDDS was found in solution at the end of the experiment (480 h, 20 days) in S_2_ and S_3_, while 45% was kept in S_1_. In any case, it is considerably higher than the sum of the solubilized metal at 480 h. Despite the large [*S,S*]-EDDS decrease in these soils, the amount of solubilized metals does not decrease in the same proportion (Figure 2). The contribution of the [*S,S*]-EDDS degradation compounds may probably aid in keeping these metals in solution, as has been previously pointed out for Zn solubilization in previous works.^9^ The lower degradation in S_1_ should be related to the lower Fe and Mn availability in the soils and solubilization by [*S,S*]-EDDS. The implications of these metals in the biodegradation of [*S,S*]-EDDS are suspected.

### 3.2 Effect of [*S,S*]-EDDS Soil Application on Plant Development and Nutrition

**Figure 4:**
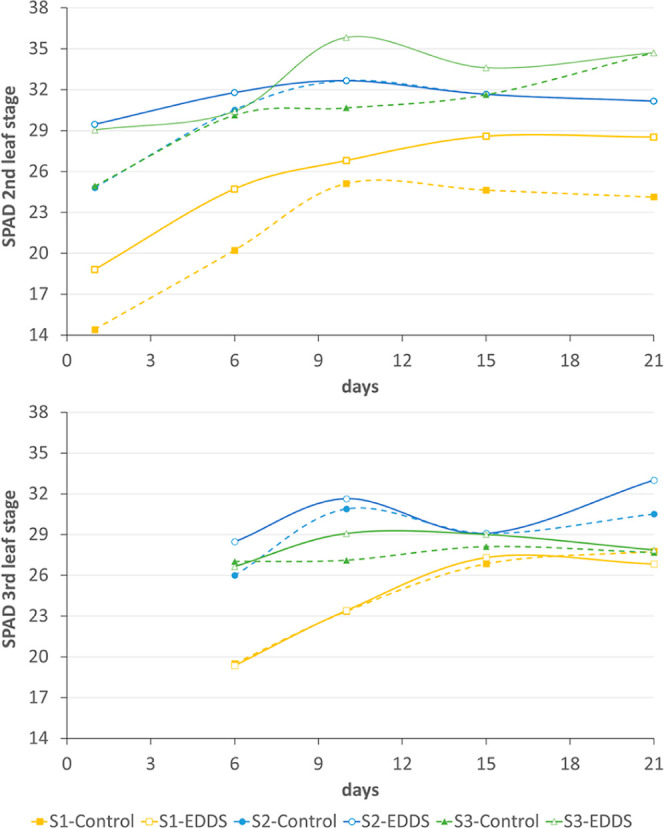
SPAD indices of the second and the third leaf stages of plants during the experiment. Dots represent means (*n* = 5).

SPAD indexes of the second and the third leaf stages were statistically analyzed for the three factors (soil, treatment, and day), showing that the application of [*S,S*]-EDDS to the soils increased the chlorophyll concentrations in the three soils. The increase occurred up to day 10 and after that, the values stabilized (see Figure 4 and Table 4). The influence of the soil type was evident in the SPAD indices, showing the plants growing in S_1_ correspond to the soil with the lowest micronutrient concentrations, and the lowest values. The effects of soil type and ligand also significantly affected the dry mass of the plants (Table 5). The application of [*S,S*]-EDDS to the soil increased the dry mass, especially in S_1_ in a range of 1.36 (stem) to 1.75-fold (leaves) as compared to control plants. Lower differences were obtained in plants grown in S_2_, where the plants were bigger in general. Only a few plants presented flowers or fruits at the sampling time; thus, no differences were found due to high data variability.

**Table 4:** Statistics for SPAD Indices at the Second and the Third Leaf Stages (Average and Standard Error Values Represented in Figure 4)

	2nd leaf stage^a^	3rd leaf stage^a^
FACTOR A: soil	***	***
FACTOR B: treatment	***	*
FACTOR C: days	***	***
A × B	***	ns
A × C	ns	ns
B × C	ns	ns
A × B × C	ns	ns

a *Significant at *P* < 0.05, **significant at *P* < 0.01, ***significant at *P* < 0.001 levels, ns not significant.

**Table 5:** Dry Mass (g plant^–1^) of Plants at the End of the Plant Experiment

soil	treatment	dry mass (g plant^–1^)			
		leaves	stem	root	flower + fruit
S_1_	[*S,S*]-EDDS	0.330 ± 0.018	0.216 ± 0.014	0.302 ± 0.059	0.031 ± 0.006
	control	0.188 ± 0.032	0.159 ± 0.162	0.180 ± 0.034	0.015 ± 0.002
S_2_	[*S,S*]-EDDS	0.308 ± 0.013	0.218 ± 0.020	0.267 ± 0.019	0.014 ± 0.006
	control	0.296 ± 0.031	0.190 ± 0.027	0.212 ± 0.026	0.015 ± 0.006
S_3_	[*S,S*]-EDDS	0.226 ± 0.041	0.162 ± 0.031	0.181 ± 0.030	0.019 ± 0.003
	control	0.182 ± 0.025	0.129 ± 0.008	0.174 ± 0.019	0.010 ± 0.003
statistics^a^					
FACTOR A: soil		**	*	ns	ns
FACTOR B: treatment		**	*	*	ns
A × B		ns	ns	ns	ns

a *Significant at *P* < 0.05, **significant at *P* < 0.01, ***significant at P < 0.001 levels, ns not significant.

**Figure 5:**
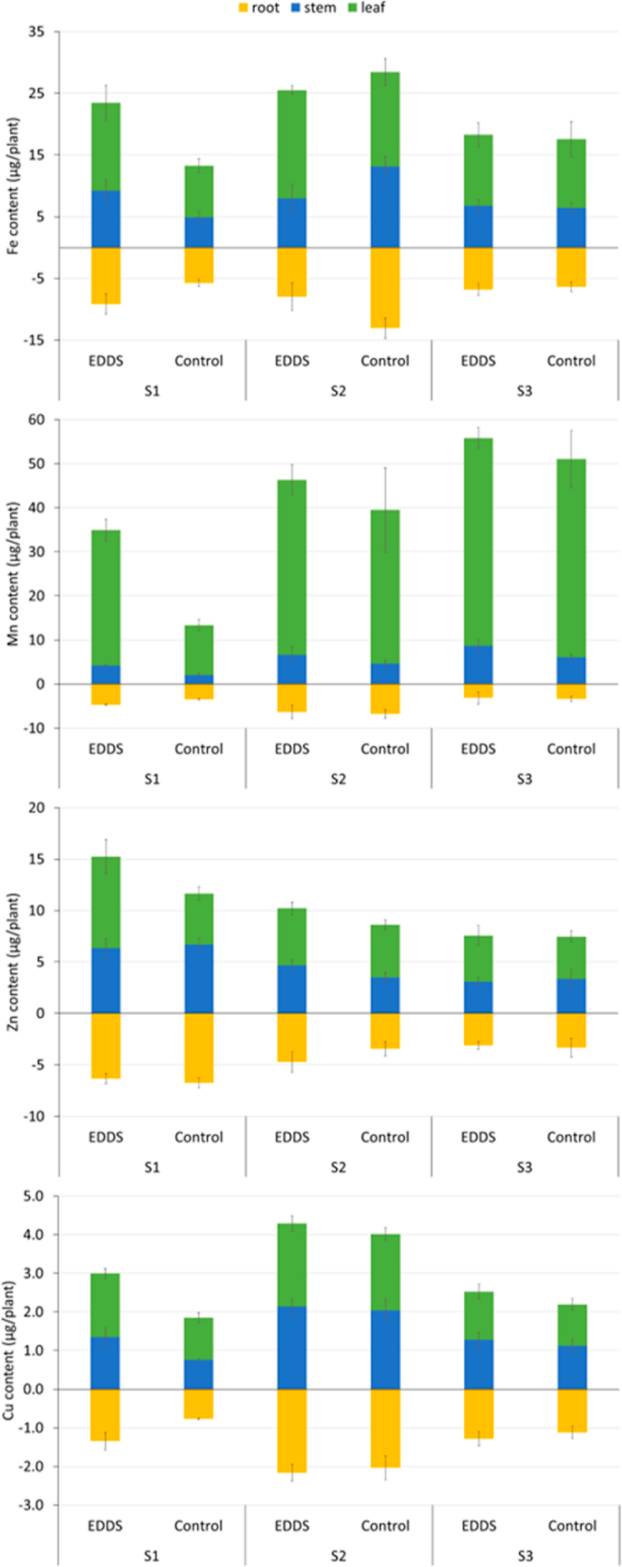
Distribution of the micronutrient content (μg plant^–1^) in different plant organs at the end of the experiment. Values are means ± SD (*n* = 5). Root content is expressed as negative values.

The effect of the [*S,S*]-EDDS addition to soil also affected the nutritional content of plants. The results for micronutrients are presented in Figure 5, and the statistical analysis is presented in Table 6. In general, the highest differences were found in S_1_. In this soil, the ligand application improved the Fe, Mn, Zn, and Cu plant content. Closer values were found in S_2_ and S_3_; in these soils, the control plants presented higher nutritional contents than those of S_1_ control plants; thus, the improvement of the nutrition is more difficult to observe.

**Table 6:** Statistics for Micronutrient Content (μg plant^–1^) in Leaves (L), Stem (S), and Root (R) of Plants at the End of the Experiment (Average and Standard Error Values Represented in Figure 5)

	Fe^a^			Mn			Zn			Cu		
	L	S	R	L	S	R	L	S	R	L	S	R
FACTOR A: soil	*	ns	*	***	**	**	*	***	***	***	***	***
FACTOR B: treatment	ns	**	ns	*	*	**	*	*	ns	*	ns	ns
A × B	*	*	ns	ns	ns	ns	*	ns	ns	ns	ns	ns

a *Significant at *P* < 0.05, **significant at *P* < 0.01, ***significant at *P* < 0.001 levels, ns not significant.

Focusing on each micronutrient, the bifactorial statistical analysis indicated that the Fe content in leaves or roots was not affected by the ligand application but by the soil type. In agreement with the significant interaction in leaves, it is observed that control plants growing in S_1_ presented lower Fe contents than the others. In this soil, the Fe content was significantly higher after the application of [*S,S*]-EDDS to the soil. It was also in this soil, where the Fe solubilization increased more continuously, pointing to longer stability (Figure 2). In terms of the Fe concentration, the values were similar for the stem and root but; for leaves, the Fe concentration was also higher for the [*S,S*]-EDDS treated plants (14.2 ± 2.8 and 8.4 ± 1.1 μg g^–1^ for [*S,S*]-EDDS and control, respectively). This fact highlights that the Fe accumulation was improved by the mass increment of the plants and Fe soil mobilization. The Fe content of plants grown in S_3_ was similar; thus, the effect due to the ligand application was not found. Fe solubilized by this ligand in the batch experiment was only significant for the first few days, decreasing rapidly after eight days. The results agree with those previously reported.

Regarding the Mn content in plants, higher differences were found again in S_1_. Control plants growing in this soil presented the lowest values in terms of content but also in concentration (a leaf concentration of 66.3 ± 9.3 μg g^–1^ for control and 93.9 ± 8.1 μg g^–1^ for treated plants). In the other soils, the Mn concentrations were higher (average Mn concentrations of 123 and 256 μg g^–1^ in S_2_ and S_3_, respectively); thus, not indicating any deficiency and impairing the observation of any Mn improvement by the [*S,S*]-EDDS application.

The Zn content of plants was significantly affected by the soil type and the treatment. In general, the Zn accumulation increased by the [*S,S*]-EDDS application, but the differences were less remarkable than in the other nutrients. On the contrary to that occurring with Fe and Mn, the S_3_ presented lower values for Zn content. This agrees with the soil analysis that indicated the very low Zn concentration available for this soil. The Zn content in leaves of plants grew in S_1_ with [*S,S*]-EDDS application being almost double that found in the rest of the plants; however, in terms of concentration, they present similar values (in the range of 17.5–28 μg g^–1^).

Attending to the Cu analysis in plants, the most remarkable differences were found again in S_1_. In this soil, the application of [*S,S*]-EDDS to the soil improved the Cu uptake by plants. In the other two soils, the total values were higher, even for the control plants; thus, the application of the ligand did not improve the Cu accumulation. This similitude was also evident in terms of the Cu concentration (average data 6.9 and 5.9 μg g^–1^ for S_2_ and S_3_, respectively).

### 3.3 Availability of Nutrients in Soils after Plant Growth

The pot soils were analyzed for the available Fe, Mn, Zn, and Cu at the end of the plant experiment. The results are shown in Table 7. Again, the effect of the soil was significant for all the micronutrients, and the effect of the treatment was only significant for Zn and Cu in the bifactorial statistical analysis. Focusing on these two micronutrients, the Zn concentration was slightly improved by the [*S,S*]-EDDS application in S_1_ and S_2_; in these soils, the highest Zn content in plants was found (Figure 5). The Cu concentrations found in the soil are coherent with the Cu contents in plants; the highest Cu concentrations in plants and soil were found in S_2_. It must be highlighted that according to the initial soil analysis S_2_ presents higher Cu concentrations in comparison to the other soils (Table 1). These results are in agreement with Tandy et al.,^34^ resulting in [*S,S*]-EDDS being a more effective extractant of Zn and Cu than EDTA.

**Table 7:** Micronutrient Concentration in Pot (μg g^–1^ Soil) Soils at the End of the Plant Experiment

soil	treatment	soil concentration (μg g^–1^soil)			
		Fe	Mn	Zn	Cu
S_1_	[*S,S*]-EDDS	0.365 ± 0.030	0.972 ± 0.140	0.353 ± 0.018	0.136 ± 0.014
	control	0.636 ± 0.052	0.578 ± 0.050	0.258 ± 0.008	0.0917 ± 0.0018
S_2_	[*S,S*]-EDDS	2.38 ± 0.05	1.84 ± 0.12	0.438 ± 0.010	1.12 ± 0.03
	control	1.71 ± 0.16	1.53 ± 0.16	0.347 ± 0.018	0.731 ± 0.056
S_3_	[*S,S*]-EDDS	0.638 ± 0.060	1.52 ± 0.23	0.364 ± 0.019	0.504 ± 0.006
	control	0.914 ± 0.042	3.02 ± 1.03	0.406 ± 0.041	0.558 ± 0.026
statistics					
FACTOR A: soil		***	*	***	***
FACTOR B: treatment		ns	ns	**	***
A × B		***	ns	**	***

The highest effect of the [*S,S*]-EDDS application in plant content was observed in S_1_. Taking a look into the remaining concentrations analyzed at the end of the experiment, the Mn, Zn, and Cu pool in the soil could promote a higher increment of the plant metal accumulation. This is not the case with Fe, where the final concentration in the soil was lower in the [*S,S*]-EDDS-treated soil than in the control soil. It must be noticed that despite this soil having a very low Fe available concentration, the plants improved the Fe nutrition when the soil was treated with the [*S,S*]-EDDS, achieving values close to the plants growing in S_2_, where the Fe concentration in soil was remarkably higher. Thus, effective Fe mobilization was done on the non-available Fe fraction by the ligand application. In previous works, the application of the [*S,S*]-EDDS chelated with Fe improved the Fe nutrition of plants growing in calcareous soil and the Zn nutrition, even better than the EDTA despite the degradation of the ligand expected.^9^ In this work, it confirmed the capacity of the metal-free ligand [*S,S*]-EDDS to solubilize Fe and other micronutrients but also revealed that the degradation compounds contributed to solubilizing and promoting plant nutrition even after the [*S,S*]-EDDS is degraded. However, studies focused on the complexing capacity or solubilization from the soil of the degraded compounds have not been found in the literature.

In summary, these results highlighted the potential use of the metal-free ligand [*S,S*]-EDDS in soils to improve the Fe nutritional status in plants, especially in sandy-clay soils with a remarkably low Fe availability, typically found in the Mediterranean areas. Despite the common method of correction of micronutrient deficiencies being the application of metal-chelated formulations, the applicability of these findings will support the direct application of the ligand to the soil to reduce the deficiencies and the possibility of its biotechnological application by the direct bacteria producer implementation to the soil. Further investigation with this approach would be needed.

## Abbreviations

*o,o*-EDDHA: ethylenediamine-*N*-*N*′bis(*o*-hydroxyphenylacetic) acid
AAS: flame atomic absorption spectrophotometry
DTPA–TEA: diethylenetriaminepentaacetic acid–triethanolamine
EDTA: ethylenediaminetetraacetic acid
HBED: *N*,*N*′-bis(2-hydroxybenzyl)ethylenediamine-*N*,*N*′-diacetic acid
HEPES: 4-(2-hydroxyethyl)-1-piperazineethanesulfonic acid
HPLC-PDA: high-performance liquid chromatography coupled to a photodiode array
[*S,S*]-EDDS: *S*,*S*-isomer of the ethylenediaminedisuccinate
S_1_: soil from Picassent
S_2_: soil from Alicante
S_3_: soil from Burgos
TBAOH: tetrabutylammonium hydroxide solution
